# The landscape of DNA methylation-mediated regulation of long non-coding RNAs in breast cancer

**DOI:** 10.18632/oncotarget.17705

**Published:** 2017-05-08

**Authors:** Chunlong Zhang, Xinyu Wang, Xuecang Li, Ning Zhao, Yihan Wang, Xiaole Han, Ce Ci, Jian Zhang, Meng Li, Yan Zhang

**Affiliations:** ^1^ Department of Medical Informatics, Daqing Campus, Harbin Medical University, Daqing, 163000, China; ^2^ College of Bioinformatics Science and Technology, Harbin Medical University, Harbin, 150081, China; ^3^ School of Life Science and Technology, Harbin Institute of Technology, Harbin, 150081, China

**Keywords:** lncRNA, DNA methylation, network, breast cancer, hallmark

## Abstract

Although systematic studies have identified a host of long non-coding RNAs (lncRNAs) which are involved in breast cancer, the knowledge about the methyla-tion-mediated dysregulation of those lncRNAs remains limited. Here, we integrated multi-omics data to analyze the methylated alteration of lncRNAs in breast invasive carcinoma (BRCA). We found that lncRNAs showed diverse methylation patterns on promoter regions in BRCA. LncRNAs were divided into two categories and four subcategories based on their promoter methylation patterns and expression levels be-tween tumor and normal samples. Through cis-regulatory analysis and gene ontology network, abnormally methylated lncRNAs were identified to be associated with can-cer regulation, proliferation or expression of transcription factors. Competing endog-enous RNA network and functional enrichment analysis of abnormally methylated lncRNAs showed that lncRNAs with different methylation patterns were involved in several hallmarks and KEGG pathways of cancers significantly. Finally, survival analysis based on mRNA modules in networks revealed that lncRNAs silenced by high methylation were associated with prognosis significantly in BRCA. This study enhances the understanding of aberrantly methylated patterns of lncRNAs and pro-vides a novel insight for identifying cancer biomarkers and potential therapeutic tar-gets in breast cancer.

## INTRODUCTION

Breast cancer is a genetically malignant tumor caused by a variety of elements involving the accumulation of genetic changes [1–3]. During the past decades, many genetic pathogenic factors have been recognized. With the study developed, besides PCGs large numbers of non-coding RNAs (ncRNAs) which were transcribed by genome sequences but not coding proteins were identified [4, 5]. MicroRNAs (miRNAs) and long non-coding RNAs (lncRNAs) are crucial directions in the researches of ncRNAs. The miRNAs inhibit the translation or regulation of target genes that carry miRNA binding sites in their 3′ untranslated regions (UTRs) [6]. Many investigations illustrated the importance of miRNAs in diseases [7]. MiRNAs are also implicated in breast cancer and many other cancers [8]. For example, the miR-200 family (miR-200a, miR-200b, miR-200c, miR-141 and miR-429) and miR-205 regulated epithelial to mesenchymal transition by targeting *ZEB1* and *SIP1* [9]. These two genes were involved in epithelial to mesenchymal transition and tumor metastasis. Yang et al. [10] proved that the overexpression of miR-346 reduced the expression of *SRCIN1* and promoted cell proliferation, colony formation, and sensitivity to Docetaxel (Doc) in breast cancer. For lncRNAs, Jadaliha et al. [11] used MALAT1 knockdown/overexpression experiments to confirm the functional significance of MALAT1 as a metastasis driver and a prognostic factor in ER negative, lymph node negative breast cancer. Zhang et al. [12] substantiated that HOTAIR was a biomarker for breast cancer. Although ncRNAs have been described with relatively definite molecular mechanisms in cancers, the regulatory mechanisms of ncRNAs in tumors were unclear especially DNA methylation of lncRNAs.

As one of the most important epigenetic modifications, DNA methylation involves the addition of a methyl group to the C5 position of cytosine residues which is catalyzed by DNA methyltransferases. For PCGs the hypermethylation in promoter inhibits the combination of transcription factors and silences cancer suppressor genes, whereas the hypomethylation in promoter activates oncogenes [2, 13]. For instance, Tang et al. [14] have found that hypomethylation of *RPTOR*, *MGRN1* and *RAPSN* led to high odds ratios (ORs) in peripheral blood DNA of breast cancer compared with normal tissues and the function of hypomethylation was validated in three independent large sample sets through MassARRAY EpiTyper assays. Yi et al. [15] have verified that overexpression of *NSUN2* by DNA hypomethylation facilitated cell proliferation, migration, and invasion in the progress and development of breast cancer. In addition, Luo et al. analyzed the relationship between *PTEN* hypermethylation and breast cancer. *PTEN* promoter hypermethylation was increased significantly in ductal carcinoma *in situ* (DCIS) and invasive ductal carcinoma (IDC) and associated with the risk of DCIS and IDC [16]. Yu et al. [17] found the promoter hypermethylation led to the silence of *RASSF2A* which was regarded as a tumor suppressor gene. Therefore aberrant DNA methylation in promoter is a prominent feature for the identification of new targets in the pathophysiology of tumors and therapeutic intervention.

Based on the research foundation of PCGs, we assumed that DNA methylation of lncRNA promoters might be an epigenetic regulator of lncRNAs expression. Several lncRNAs have demonstrated the hypothesis. For instance, the hypomethylation of AFAP1-AS1 in Barrett's esophagus and esophageal adenocarcinoma caused the overexpression of AFAP1-AS and affected the proliferation and colony-forming ability [18]. Aberrant DNA hypermethylation downregulated the expression of SOX21-AS1 and low expression of SOX21-AS1 might be an adverse prognostic biomarker in oral cancer [19]. However, these researches only analyzed the DNA methylation of few specific lncRNAs but did not identify the DNA methylation pattern of lncRNAs systematically. Liao et al. [20] analyzed the DNA methylation models of lncRNA promoter in colorectal cancer. They found the hypermethylation of lncRNA promoter offered a new clue for the biological researchers to further understand the function of lncRNAs in colorectal cancer. Zhi et al. [21] created a novel re-annotation strategy to display the DNA methylation patterns of lncRNAs in pan-cancers. But the two studies did not assess how DNA methylation regulated the expression of lncRNAs accurately. They only used few methods to verify the function of lncRNAs.

With the improvement of high-throughput sequencing technology, multitudinous data such as RNA-seq and HumanMethylation450 BeadChip (450K) have been applied for the analysis of cancers [22, 23]. High throughput multi-omics data has facilitated the development of large-scale identification and systemic analysis of novel cancer biomarkers. In this study, a new strategy was raised to observe the DNA methylation patterns of lncRNAs systematically based on genome-wide lncRNA expression, gene expression and DNA methylation profiles in BRCA from The Cancer Genome Atlas (TCGA) project. Firstly, we re-annotated the promoters of lncRNAs and obtained 137 high methylated lncRNAs (HMLncs) and 101 low methylated lncRNAs (LMLncs). Then cis-regulatory function enrichment analysis and gene ontology (GO) term network were carried out to predict the function of lncRNAs. HMLncs and LMLncs mainly influenced regulation (HMLncs: 126, LMLncs: 118). Moreover, LMLncs also influenced proliferation. Next, using the investigation of the competing endogenous RNA (ceRNA) networks, we discovered that lncRNAs classified by DNA methylation were enriched in different types of cancer hallmarks, and validated the impact of DNA methylation for lncRNAs. Finally, through the survival analysis, we identified high-down module associated with prognosis in BRCA. The study presents the methylated regulation mechanism of lncRNAs in BRCA and provided potential cancer biomarkers for diagnosis and treatment.

## RESULTS

### DNA methylation pattern in lncRNA promoters

To assess patterns of lncRNA, the DNA methylation, lncRNA expression, mRNA expression and clinical data of breast invasive carcinoma (BRCA) were used in this study. The quantity of samples for each profile was in Table 1.

**Table 1:** The number of samples in each type of data

Data Type	Tumor	Normal
DNA methylation	529	97
lncRNA expression	529	105
mRNA expression	529	113
Clinical data from TCGA	502	-
Clinical data from GSE2034	286	-
Clinical data from GSE2990	187	-
Clinical data from GSE7390	198	-

**Figure 1:**
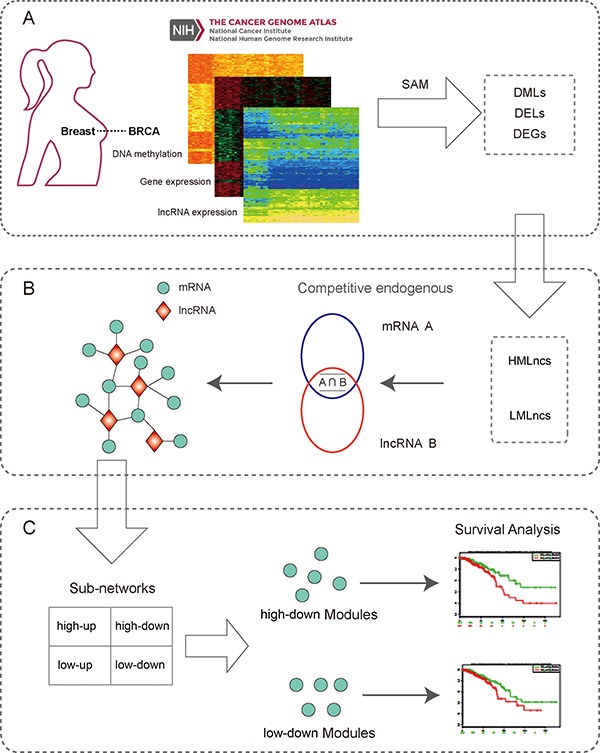
The workflow of our study (**A**) Data source and molecular filter. Expression and methylation data were downloaded from The Cancer Genome Atlas database. Then differential genes and lncRNAs were extracted by SAM method. (**B**) The construction of lncRNA-mRNA ceRNA network. The intersection of DMLs and DELs were re-classified into HMLncs and LMLncs based on the pattern of methylation. The competitive endogenous mRNAs of these lncRNAs were obtained to construct the ceRNA network. Blue and red circles are the miRNAs interacted with mRNA A and lncRNA B, respectively. (**C**) The analysis of lncRNAs in different networks. Four sub-networks were extracted from the background network. Sub-networks were analyzed by various methods such as function enrichment analysis, hallmark analysis and survival analysis to validate the effect of DNA methylation to lncRNAs. (DML: Differentially Methylated LncRNA, DEL: Differentially Expressed LncRNA, DEG: Differentially Expressed Gene, HMLnc: high methylated LncRNA, LMLnc: low methylated LncRNA, PCG: Protein Coding Gene).

After the profiles preprocessed, differentially methylated lncRNAs (DMLs), differentially expressed lncRNAs (DELs) and differentially expressed genes (DEGs) were recognized respectively between tumor and normal samples (Figure 1A). To estimate the importance of DNA methylation for lncRNAs, lncRNAs were divided into two categories based on the mean methylation level in promoter and expression of lncRNAs. Both the categories were differentially expressed lncRNAs. The first category of lncRNAs were the high methylated lncRNAs (HMLncs) that DNA methylation level in promoter was up-regulated compared with normal samples. The other category of lncRNAs were the low methylated lncRNAs (LMLncs) that DNA methylation level in promoter was down-regulated compared with normal samples.

Next we used GREAT, HMDD and ceRNA networks to identify the influence of lncRNAs in BRCA. Four sub-networks were extracted from the background ceRNA network. Then two modules were got from high-down and low-down network. To evaluate the association of the two modules with patient prognosis we used clinical data from TCGA and three sample sets from GEO to do survival analysis to verify the reliability of our modules (Figure 1B and 1C).

We regarded the region 2kb upstream from TSS as the promoter of lncRNA. In this study, 18,772 probes were located in most lncRNA promoters (87.53%, 3,459). The average value of DNA methylation probes within one lncRNA promoter was computed as the DNA methylation level of the lncRNA. The DNA methylation levels in promoters had a different distribution between tumor and normal samples (Kolmogorov-Smirnov (K-S) test, *P* = 7.15e-05). By comparing the DNA methylation and gene expression between tumor and normal samples, we identified 743 DMLs and 3206 DELs. This result indicated that lncRNAs exhibited differentially methylated and expressed patterns between tumor and normal samples.

**Figure 2:**
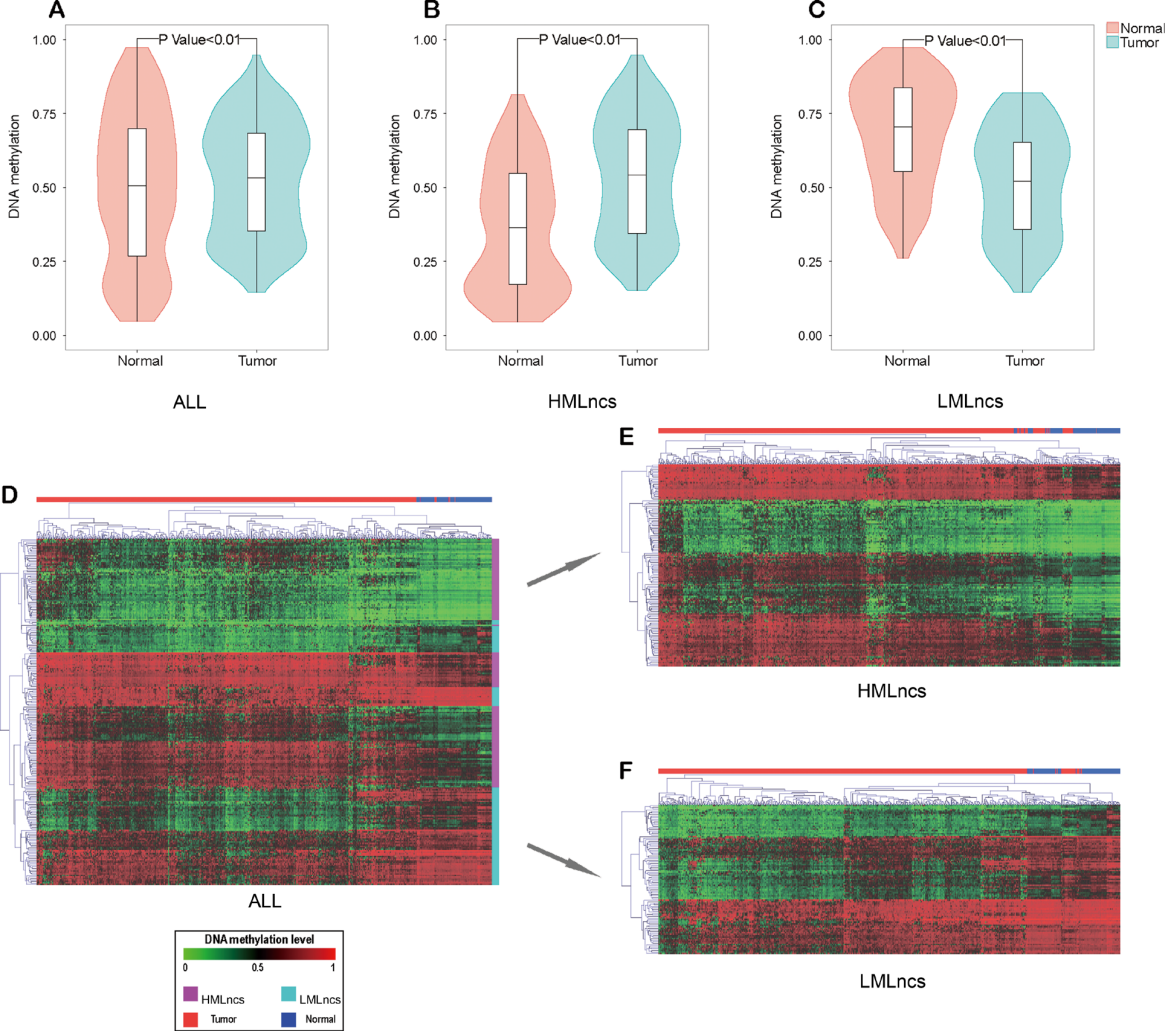
The DNA methylation patterns of tumors and normal samples (**A**) The methylation level of all differential lncRNAs (HMLncs + LMLncs). (**B**) The methylation level of HMLncs. (**C**) The methylation level of LMLncs. (**D**) The bidirectional hierarchical cluster of all differential lncRNAs (HMLncs + LMLncs). (**E**) The bidirectional hierarchical cluster of HMLncs. (**F**) The bidirectional hierarchical cluster of LMLncs.

Then 238 lncRNAs (137 HMLncs and 101 LMLncs) that showed both differential methylation and differential expression were picked for the further analysis of lncRNAs. Aberrant DNA methyltransferases might cause the chromosome abnormality and tumor development, and cancer cells usually showed higher methylation than normal cells in promoters of PCGs [24]. We found DNA methylation level in lncRNA promoter showed the similar pattern with that in PCG promoter and was high methylation in tumor samples. Then DNA methylation levels in tumor and normal samples were used to assess the statistical difference with the SAM method based on all 238 lncRNAs, 137 HMLncs and 101 LMLncs, respectively. The three lncRNA sets were all differential (*P* < =0.01) (Figure 2A, 2B and 2C). The overall DNA methylation level in promoter tended to rise and exceeded 0.5 (Figure 2A). Moreover bidirectional hierarchical clustering analysis using DNA methylation level indicated that the 238 lncRNAs divided the tumor and normal samples obviously. HMLncs and LMLncs both could be clustered into several groups. The result suggested that 238 DMLs in tumors had similar methylation patterns, and HMLncs and LMLncs also displayed the consistency of DNA methylation levels in tumors respectively (Figure 2D, 2E and 2F).

### The function interpretation of cis-regulatory regions for lncRNA

Previous studies proved that differentially methylated lncRNAs might be involved in DNA repair, cell apoptosis, cell cycle and many other cancer-related functions [20].

**Figure 3:**
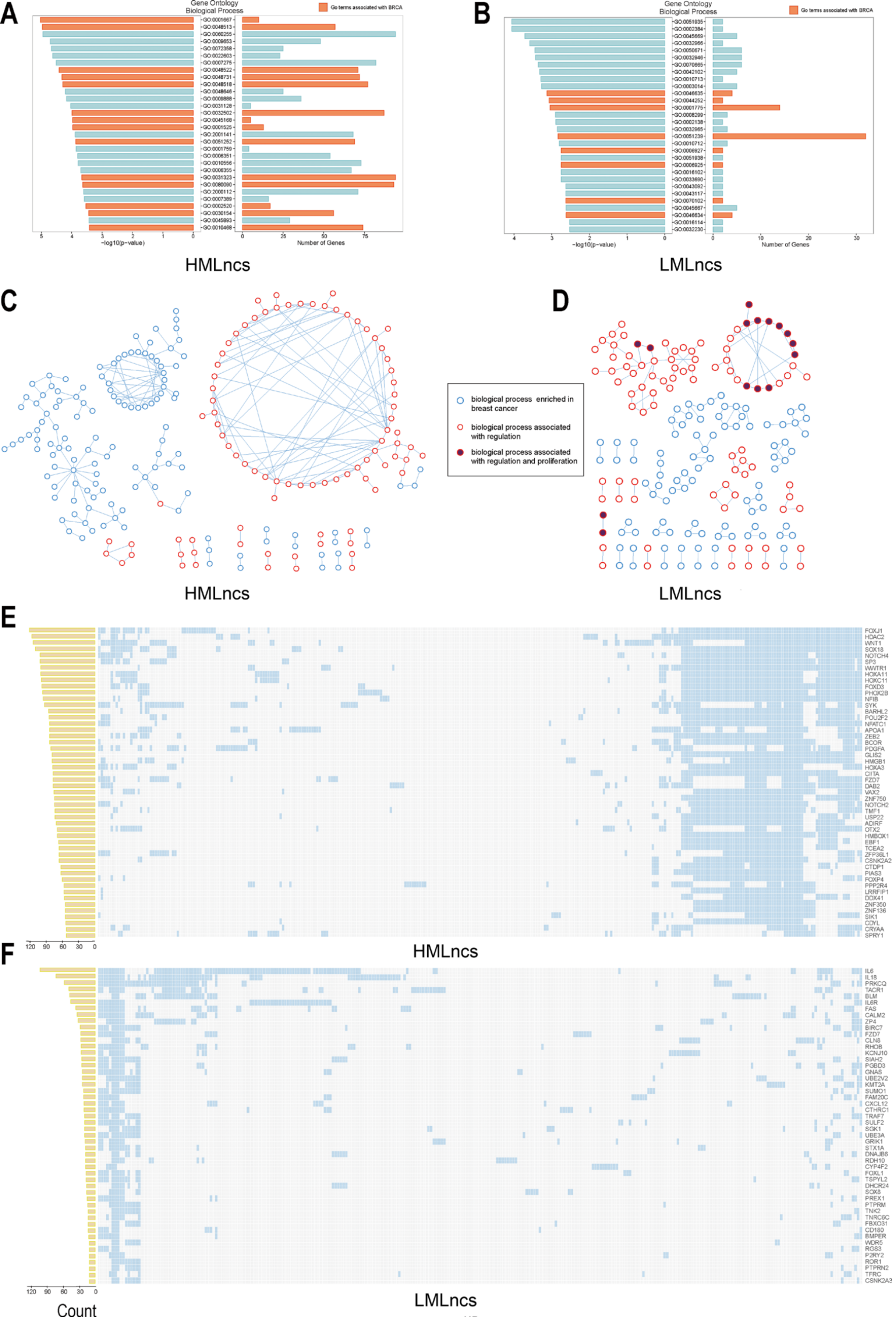
The cis-regulatory function of HMLncs and LMLncs (**A** and **B**) The top-30 ranking GO terms of HMLncs and LMLncs based on *p*-values, respectively. The orange bars represented the GO terms associated with breast cancer. (**C** and **D**) The GO term networks. The nodes represented the GO terms enriched significantly. (**E** and **F**) The frequency of occurrence of genes in GO terms of HMLncs and LMLncs. Each row represented a gene. And each column was a GO term. The genes were selected if they were regulated by most lncRNAs (top 50 genes). The bar plot at the left side was the count of GO terms of each gene.

In order to estimate whether lncRNAs classified by DNA methylation influenced tumor progress through cis-regulatory mechanism, a nearest strategy was applied to analyze the function of lncRNAs through the tool GREAT. The tool GREAT integrated annotations from 20 ontologies including gene ontology, Human Phenotype, Disease Ontology, MSigDB Cancer Neighborhood, PANTHER Pathway and so on. Indeed, the lncRNAs were significantly enriched in many ontologies (*p* < =0.05) (Figure 3). For example, HMLncs were mainly enriched in ameboidal cell migration (GO:0001667), organ development (GO:0048513), positive regulation of cellular process (GO:0048522) and system development (GO:0048731) for gene ontology. LMLncs were mainly enriched in positive regulation of alpha-beta T cell activation (GO:0046635), negative regulation of multicellular organismal metabolic process (GO:0044252), cell activation (GO:0001775) and regulation of multicellular organismal process (GO:0051239) for gene ontology. Cell-cell signaling, cell process, metabolism and angiogenesis were deeply correlated with the progression of cancers [25, 26]. Moreover, GO terms (top 30 rank of *p-value*) in HMLncs exhibited much stronger relevance than those in LMLncs according to *p-value* and gene number (Figure 3A and 3B). Besides, we also summarized the functions of disease ontology, MSigDB pathway and PANTHER pathway. HMLncs were enriched in malignant neoplasm of breast and focal adhesion, and LMLncs were enriched in mammary cancer, STAT3 pathway and Wnt signaling pathway [27–30] (Supplementary Table 1).

To illustrate the relationship among GO terms enriched by lncRNAs, the GO terms were inputted into GO term network. The HMLncs network had three large connected components (nodes > 20). The largest one included 66 GO terms and 63 of them were related to regulation (Figure 3C). The LMLncs network had two large connected components (nodes > 20). They included 38 and 22 GO terms, respectively. And all of the GO terms in two large connected components were related to regulation. Besides, 13 GO terms (59.09% of second large connected component) were related to proliferation (Figure 3D). The results suggested that lncRNAs mainly interfered in the regulation and proliferation of BRCA, and these biological processes with similar functions often kept the strong connectedness with each other.

To evaluate which PCGs were cis-regulated by lncRNAs frequently, we counted the number of PCGs in the result of GREAT enrichment analysis (biological process) for HMLncs and LMLncs. The genes were ranked by the frequency of enrichments in biological process. Of the top 10 genes in HMLncs, nine were demonstrated to be associated with BRCA: *FOXJ1* [31], *HDAC2* [32], *WNT1* [33], *SOX18* [34], *NOTCH4* [35], *SP3* [36], *HOXA11* [37], *HOXC11* [38] and *FOXD3* [39]. In addition, there were six genes coding transcription factors in the top 10 genes (*FOXJ1*, *SOX18*, *SP3*, *HOXA11*, *HOXC11* and *FOXD3*) (Figure 3E). *FOXJ1* and *FOXD3* were the members of forkhead box (FOX) and genes in FOX family played key roles in many cancer-related biological processes, such as metastasis, development, organization differentiation, cell proliferation, cell apoptosis, cell migration, invasion, and longevity [40]. *HOXA11*, and *HOXC11* were the members of homeobox family and the two genes were validated to be correlated with the survival of BRCA [37, 38].

In conclusion, these HMLncs tended to cis-regulate genes which coded transcription factors and lead to the dysregulation of transcription in BRCA.

For LMLncs, Interleukins were a group of cytokines which regulated the immune systems and many autoimmune diseases or immune deficiency were lack of them [41]. Interleukin-6 (*IL6*, GO term count=103), interleukin-18 (*IL18*, GO term count=73) and Interleukin-6 receptor (*IL6R*, GO term count=46) in top 10 genes of LMLncs (Figure 3F) have important roles in inflammation and many biological processes such as enhancing the power of tumor cell apoptosis, inhibiting tumorigenesis and inhibiting tumor angiogenesis [42, 43].

### The identification of cancer-related hallmarks in ceRNA network

To analyze how DNA methylation regulated the expression of lncRNAs, we further separated HMLncs and LMLncs into four groups: high-up group (high methylation and up-regulated lncRNA expression), high-down group (high methylation and down-regulated lncRNA expression), low-up group (low methylation and up-regulated lncRNA expression) and low-down group (low methylation and down-regulated lncRNA expression).

**Figure 4:**
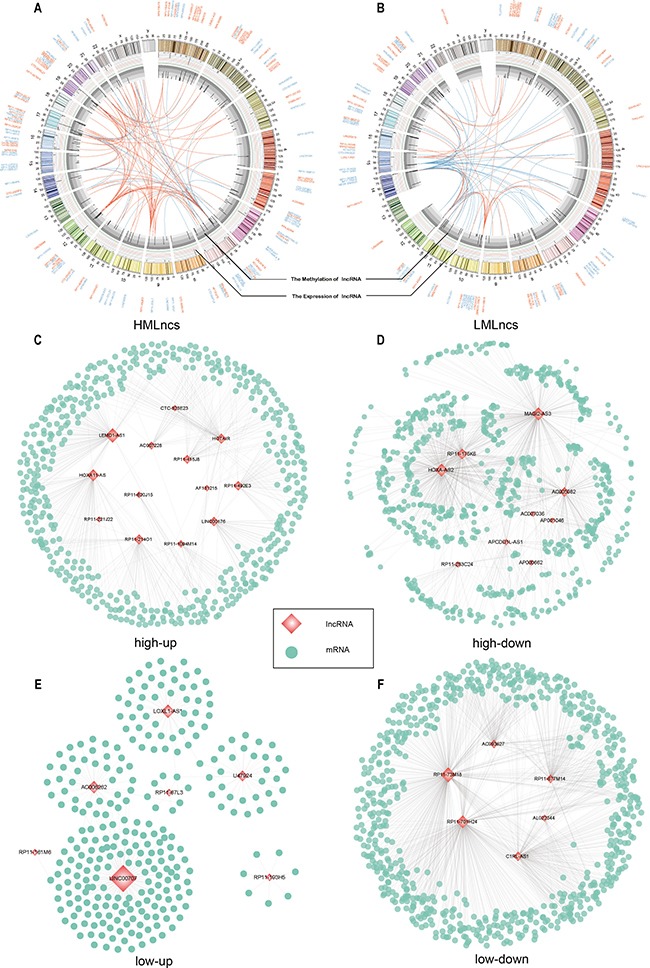
The lncRNA-mRNA ceRNA networks (**A** and **B**) The relationship between lncRNAs and miRNAs. The lncRNA names were written in the outer circle. The bar plots in the middle circle represented the methylation level and expression level of lncRNAs, respectively. The inner lines represented the interactions between lncRNAs and miRNAs. The orange color represented the up-regulated lncRNA expression. The blue color represented the down-regulated lncRNA expression. (**C, D, E** and **F**) The lncRNA-mRNA ceRNA sub-networks. The node size of lncRNAs was correlated with degree.

Many studies have illustrated that lncRNAs often combined with the target miRNAs to regulate the expression of mRNAs [44, 45]. To measure the potential tumorigenic role of lncRNAs through miR-mediated interaction in BRCA, we used DEGs and DELs to construct a background network and regarded the four groups mentioned above as seeds to mine four sub lncRNA-mRNA ceRNA networks: high-up network, high-down network, low-up network and low-down network (Table 2 and Figure 4). The lncRNAs interacted with many PCGs and had high degrees in these ceRNA networks. Besides, although the number of seed lncRNAs in high-down and low-down network were not as many as in the other two networks, the lncRNAs had the larger average degrees (high-up: 297, high-down: 504, low-up: 300, low-down: 835) (Table 2). This result suggested that down-regulated lncRNAs mediated more genes and were more valuable than up-regulated lncRNAs in BRCA.

**Table 2:** The interaction information of four groups

	lncRNAs	miRNAs	mRNAs	interaction pairs
high-up	13	101	469	558
high-down	9	51	728	1,231
low-up	7	58	277	295
low-down	6	83	687	1,129

In order to evaluate the function of four groups of lncRNAs, the PCGs in four networks were inputted into DAVID (https://david.ncifcrf.gov/) to make the function enrichment analysis (Supplementary Table 2).

Although the biological processes of tumors were especially complex, the intricacy of tumors could be simplified and represented by some cancer-related hallmarks which influenced tumor development and metabolism [46]. These hallmarks offered a new direction for understanding the mechanism of cancers.

**Figure 5:**
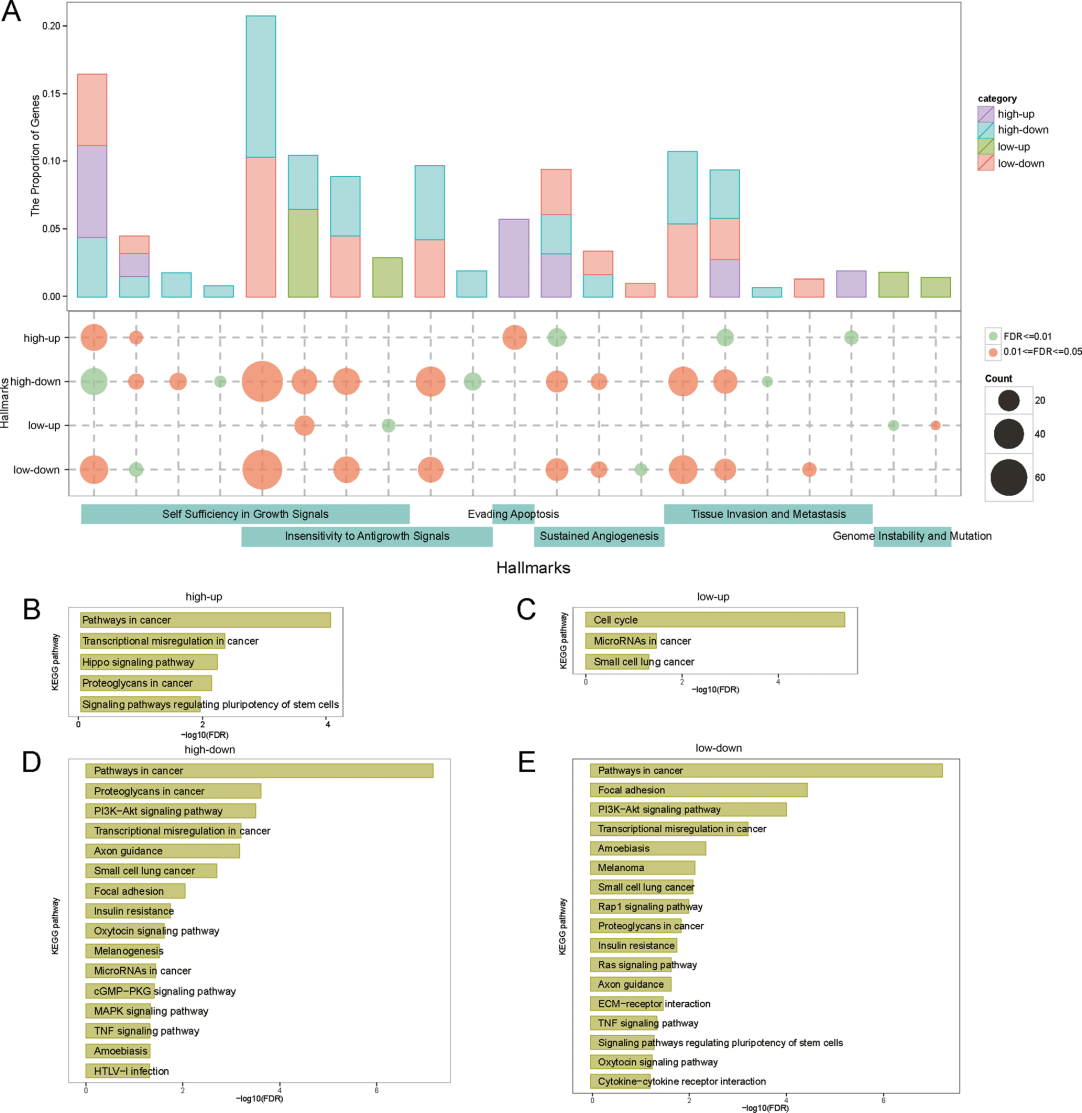
The function enrichment of mRNAs Each column represented a hallmark. (**A**) The distribution of hallmarks. The top panel was the proportion of genes: (genes enriched in hallmarks in ceRNA network). In the middle panel, the node size was the number of genes enriched in hallmarks. The bottom bar represented the classification of hallmarks. (**B, C, D** and **E**) The significant KEGG pathways.

Function enrichment analysis revealed that genes in four networks were enriched in the hallmarks of cancers significantly (Figure 5A) [47]. The genes were mainly enriched in six kinds of hallmarks (21 biological processes): “*Self Sufficiency in Growth Signals*”, “*Insensitivity to Antigrowth Signals*”, “*Evading Apoptosis*”, “*Sustained Angiogenesis*”, “*Tissue Invasion and Metastasis*” and “*Genome Instability and Mutation*”. In these hallmarks, “*Self Sufficiency in Growth Signals*” and “*Insensitivity to Antigrowth Signals*” covered the most genes and were the most affected hallmarks in BRCA. Another interesting observation was that these two kinds of hallmarks shared four biological processes (“*signal transduction*”, “*cell proliferation*”, “*intracellular signal transduction*” and “*regulation of cell cycle*”). Besides, there were six biological processes involved in the proliferation functions and this demonstrated their key roles in the development of BRCA.

An overall discovery of the function profiles of ceRNA networks also showed some common functions in different networks. For example, three networks (high-up, high-down, low-down) shared four biological processes (“*positive regulation of endothelial cell proliferation*”, “*positive regulation of mesenchymal cell proliferation*”, “*angiogenesis*” and “p*ositive regulation of cell migration*”) and these biological processes were the common characteristics of tumors. Most cancer cells owned the capacity of constant proliferation. Normal tissues always mastered the production and release of growth-promoting signals, but cancer cells disturbed the signals and led to the abnormity of tissues [48]. Cancer cells needed nutrients to sustain its growth and evacuate metabolic wastes, so they depended on the process of angiogenesis [49].

Moreover we found that the high-down and low-down network regulated 14 (66.67%) and 12 (57.14%) biological processes and almost covered all kinds of hallmarks. This implied the importance and high coverage of the down-regulated lncRNAs in the development of BRCA. In addition, the low-up network regulated “*Genome Instability and Mutation*” specifically, but other three networks were not related to this hallmark.

The four networks were also enriched in many cancer-related KEGG pathways (Figure 5B–5E). Some common KEGG pathways located in three of the four networks were identified: “*Pathways in cancer*”, “*Transcriptional misregulation in cancer*”, “*Proteoglycans in cancer*” and “*Small cell lung cancer*”. “*Pathways in cancer*” was the most significant pathway (FDR from 2.01e-06 to 8.32e-03) and was consisted of many other cancer-related pathways including “*Wnt signaling pathway*”, “*mapk signaling pathway*” and “*vega signaling pathway*” [50]. Proteoglycans in the tumor microenvironment bound to numerous matrix molecules, growth factors and inflammatory mediators thus influencing the development of cancer [51]. “*Small cell lung cancer*” also had high frequency in different networks. Our previous study has demonstrated that BRCA and lung cancer kept the strong methylation correlation [13]. We found high-down and low-down network were enriched in more KEGG pathways than the other two networks, and the results of FDR were more significantly as well (Figure 5D and 5E). Most of these pathways were associated with cancers such as “*PI3K-Akt signaling pathway*”, “*Axon guidance*” and “*TNF signaling pathway*” [52–54]. Furthermore, high-down and low-up network controlled the pathway “*microRNAs in cancer*”. The networks were filtered based on the ceRNA relationship and this result verified the reliability of our ceRNA networks.

In total, 93 genes in high-up network were enriched in hallmarks and KEGG pathways. The lncRNA LEMD1-AS1 interacted with 122 genes and 28 genes were enriched in hallmarks and KEGG pathways, and overlapped 30.11% of 93 genes. 289 genes in high-down network were enriched in hallmarks and KEGG pathways. The lncRNA MAGI2-AS3 interacted with 335 genes and 135 genes were enriched in hallmarks and KEGG pathways, and overlapped 46.71% of 289 genes. 39 genes in low-up network were enriched in hallmarks and KEGG pathways. The lncRNA LINC00707 interacted with 154 genes and 28 genes were enriched in hallmarks and KEGG pathways, and overlapped 71.8% of 39 genes. 246 genes in low-down network were enriched in hallmarks and KEGG pathways. The lncRNA RP11-73M18 interacted with 399 genes and 161 genes were enriched in hallmarks and KEGG pathways, and overlapped 40.35% of 246 genes. The lncRNAs with high degrees tended to interact with cancer-related genes, furthermore lncRNAs in high-down and low-up network had more significant coverage of cancer genes compared with other networks.

As the core link between mRNAs and lncRNAs, miRNAs played key role in development of BRCA. Therefore, miRNA enrichment analysis was used to confirm the function of ceRNA networks. Then the hypergeometric test (Materials and Methods) was used to estimate the function of miRNAs which were interacted with four lncRNA groups, respectively. The miRNAs in high-down group was involved in “*Breast Neoplasms*” significantly (*p* = 0.0366), but the other three groups were not (Supplementary Table 3). Previous studies have demonstrated that the high methylation in promoter inhibited downstream expression of lncRNA, and the silence of lncRNAs often played an important role in the progress of cancers [19]. In high-down group, 28 miRNAs were annotated in the breast neoplasms. Then the 28 miRNAs was mapped into the miRNA family information. Nine miRNAs (hsa-mir-302a, hsa-mir-302b, hsa-mir-302c, hsa-mir-302d, hsa-mir-373, hsa-mir-520a, hsa-mir-520b, hsa-mir-520c and hsa-mir-520d) were belonged to “miR-302-3p/372-3p/373” and “miR-520” families, respectively. All miRNAs have been demonstrated to be associated with BRCA through literatures by HMDD.

### The identification of clinically relevant modules in ceRNA networks

Wu et al. [55] discovered that it might be possible to improve the survival quality of cancer patients by finding and repairing survival-related gene signatures other than a single gene, and hub genes also played important roles in networks. Therefore, we extracted hub PCGs of the four networks as module to do the survival analysis. However, only high-down and low-down network had the hub PCGs (high-down: seven hub PCGs (*NFAT5*, *QKI*, *SCHIP1*, *PRRG1*, *FOXN3*, *SACS* and *NFIB*), low-down: six hub PCGs (*FOXP2*, *QKI*, *ARHGEF10*, *PTPN14*, *SPRY2* and *DPYSL2*)). Next, a multivariate survival analysis based on the two classes of hub PCG modules was performed to estimate the power of predicting survival status in BRCA.

**Figure 6:**
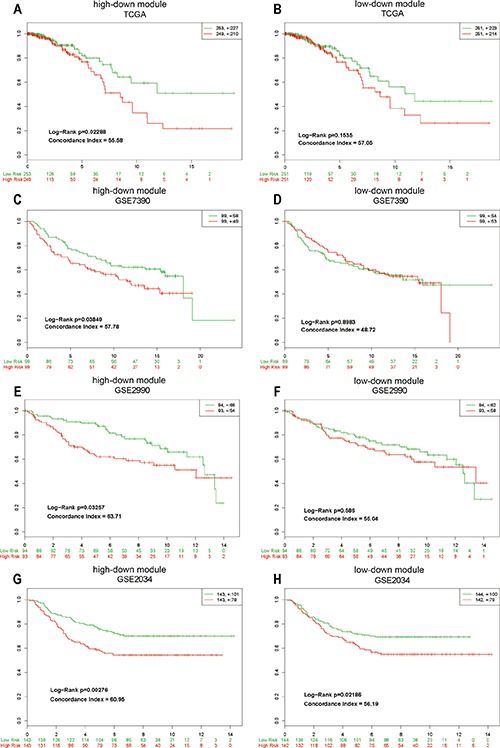
The survival analysis of high-down and low-down modules “+” represented lost to follow-up patients. The legend in the top right corner represented the total number and lost to follow-up number of patients. The green and red digitals on the bottom represented number of living patients at that time. (**A** and **B**) The survival analysis in TCGA data set. (**C–H**) The survival analysis in GEO data sets.

The high-down module was associated with the survival time in BRCA and distinguished patients into high-risk and low-risk group significantly (Log-rank test, *p* = 0.01474), but the low-down module could not (Log-rank test, *p* = 0.06716) (Figure 6A and 6B). However, it was unclear whether the high-down module had stable power of classifying the patient subtypes in BRCA significantly.

To validate the predictive power of high-down module, we did survival analysis in another three independent data sets. As a result, the high-down module kept stable separating capacity for patient survival (GSE2034: *p* = 2.76e-03, GSE2990: *p* =3.26e-02, GSE7390: *p* = 3.85e-02), but the low-down module was only significant in GSE2034 (*p* = 2.19e-02) (Figure 6C–6H). Our observation might be rewarding to separate high-risk from low-risk patients and provide a new clinical application for the prognostic and surgical management of BRCA.

## DISCUSSION

LncRNAs have been demonstrated to be associated with the development and progress of breast cancer. For instance, Xue et al. [56] has found that the overexpression of HOTAIR increased the breast cancer cell proliferation, and its depletion could contribute to the treatment of breast cancer. DNA methylation in promoter always suppresses the combination of transcription factors with DNA chain and decreases the transcription of downstream, so the expression of genes is reduced [2]. It has been recognized that changes in DNA methylation may in part be disruption of the regulatory control of specific promoter usage in cancers. Therefore the dysregulation of lncRNAs in breast cancer may be regulated by DNA methylation in promoter.

In this study, we characterized the changes in DNA methylation of lncRNA promoters in BRCA. We integrated the DNA methylation, lncRNA expression and mRNA expression profiles from TCGA and TANRIC to investigate the methylation of lncRNAs. Our research showed that a large number of lncRNAs were epigenetically deregulated in promoters. We divided the lncRNAs into different groups based on the high methylation and low methylation, and explored their biological and clinical relationship with BRCA. The result indicated that HMLncs and LMLncs cis-regulated cancer-related biological processes (Figure 3). HMLncs were enriched in 126 biological processes associated with regulation function and LMLncs were enriched in 118 biological processes associated with regulation function. Besides LMLncs were enriched in 18 biological processes associated with proliferation. In GO term networks, regulation functions and proliferation functions had the largest connected component and strong combination with each other. Moreover previous studies have shown that lncRNAs regulated PCG activity through the cis-regulation in two modes. The first mode was that the lncRNA products regulated the activity of chromosome through recruiting epigenetic modifiers. The second mode was that the transcription of lncRNAs through PCG promoters or a *cis-*regulatory element (RE) could influence the expression of PCGs [57–60]. The result showed that epigenetically dysregulated lncRNAs were always located near the genes correlated with the regulation and proliferation function. Moreover we found many high methylated lncRNAs cis-regulated PCGs which coded transcription factors such as *FOXJ1*, *SOX18*, *SP3*, *HOXA11*, *HOXC11* and *FOXD3*. In molecular biology, transcription factor could regulate the rate transcription of genetic information from DNA to mRNA by biding to a specific DNA sequence. The dysregulation of transcription factor will lead to the abnormal expression of genes near that sequence[61]. This observation suggests that high methylated lncRNAs modulate BRCA through influencing the expression of transcription factors, which provides a foundation for the further analysis of lncRNA dysregulation in BRCA.

Although we have confirmed the role of lncRNAs through cis-regulation, lncRNAs have other ways regulating cancers. Competing endogenesis relationship is another mode that lncRNAs influence the expression of mRNAs by binding to the target miRNAs of mRNAs. To predict the function of lncRNAs, we constructed ceRNA networks through four groups which were classified based on the patterns of DNA methylation and lncRNA expression. The structure of ceRNA networks showed that although high-down and low-down network consisted of less lncRNAs, they attracted more mRNAs (high-down: 728, low-down: 687) than the other two networks (Figure 4) (Table 2). Because of the positive correlation between mRNA and lncRNA, the expression of mRNAs was always inhibited. This result suggests that the majority of differentially expressed mRNA was down-regulated in BRCA.

Tumorigenesis is a complex and dynamic biological process that is controlled by multiple elements of genetic and epigenetic variations. In this study, we found that competing endogenesis relationship regulated key components of cancer-associated hallmarks and KEGG pathways [46]. Four biological processes (“*positive regulation of endothelial cell proliferation*”, “*positive regulation of mesenchymal cell proliferation*”, “*angiogenesis*” and “*positive regulation of cell migration*”) in hallmarks and three KEGG pathways (“*Pathways in cancer*”, “*Transcriptional misregulation in cancer*”, “*Proteoglycans in cancer*” and “*Small cell lung cancer*”) were identified in three lncRNA networks as the common characteristics of tumors, and previous studies have demonstrated the relationship between these functions and cancers [13, 47, 50, 51]. These results suggest the mRNAs regulated by lncRNAs could modulate the progress and development of BRCA. Moreover the high-down and low-down network also controlled more hallmarks and KEGG pathways (approximately 10 hallmarks and KEGG pathways) than high-up and low-up network and had the larger coverage of genes enriched in hallmarks and KEGG pathways (Figure 5). Moreover most of these hallmarks and KEGG pathways were associated with BRCA such as “*PI3K-Akt signaling pathway”, “Transcriptional misregulation in cancer”, “Proteoglycans in cancer*” and so on. These finding further validated the importance of down-regulated lncRNAs in BRCA. “MicroRNAs in cancer” was also enriched significantly. MiRNAs were regarded as the important element connecting the function between mRNA and lncRNA and the dysregulation of miRNAs might lead to abnormal expression of mRNAs and lncRNAs in diseases [62]. Next the miRNAs interacted with lncRNAs were used to assess the function of lncRNAs. Only high-down network was enriched in “*Breast Neoplasms*” significantly. We thought the lncRNAs down-regulated by high methylation may be more important, and also demonstrate the role of miRNA regulators in BRCA.

Finally, in order to estimate the relationship between clinical prognosis and mRNA modules, the survival analysis was used to evaluate the function of different lncRNA networks. The hub PCG module in high-down network divided patients into high-risk group and low-risk group significantly (*P* < = 0.01), but low-down PCG module could not. Next we applied three sample sets of BRCA from GEO database to verify the reliability of our finding. The result showed that high-down module divided the patients significantly in three sets (GSE2034: *p* = 2.76e-03, GSE2990: *p* = 3.26e-02, GSE7390: *p* = 3.85e-02), but low-down module was only working in one set (GSE2034: *p* = 2.19e-02) (Figure 6). From the survival analysis, the lncRNAs silenced by high methylation in promoter have been demonstrated to be potential roles in the prognosis of BRCA, and might be further evaluated for use as cancer biomarkers and potential therapeutic targets. At last, because of lack of hub PCGs in low-up module and high-up module we couldn't demonstrate the function of them for survival status.

In summary, our integration analysis of multiple omics data sets is demonstrated to identify epigenetically abnormal lncRNAs and assess the importance of high methylation for function of lncRNAs in BRCA.

The present study only analyzes the epigenetically dysregulated lncRNAs based on DNA methylation in BRCA, but recent studies have demonstrated that the N^6^-methyladenosine (m^6^A) as the RNA post-transcriptional modifications also influences the function of RNAs. Because of the lack of data for m^6^A in breast cancer, we didn't analyze the relationship between m^6^A and lncRNA [63]. In the future we hope that growing omics data in different cancers will be analyzed and biological experiments will be performed to demonstrate our study.

## MATERIALS AND METHODS

### Data source

Patient clinical data, methylation data and RNA-seq data were downloaded from TCGA (http://cancergenome.nih.gov/). Molecular data from the following platforms were used in our study. DNA methylation data was Infinium 450k arrays (Level 3) and RNA-seq data (Level 3) was RNASeqV2. The lncRNA data were obtained from TANRIC database (http://ibl.mdanderson.org/tanric/design/basic/index.html) [64]. We selected 529 tumor samples of which DNA methylation data, RNA-seq data and lncRNA data were all available.

The annotation file (gencode.v19.long_noncoding_RNAs) for lncRNAs was derived from GENCODE (http://www.gencodegenes.org/). The interaction pairs of mRNA-miRNA (386 miRNAs, 13,802 mRNAs and 423,975 interaction pairs) and lncRNA-miRNA (277 miRNAs, 1,127 lncRNAs and 10,212 interaction pairs) were downloaded from the starBase V2.0 database (http://starbase.sysu.edu.cn/) [65].

The information of transcription factors was downloaded from the Animal Transcription Factor Database (AnimalTFDB, http://www.bioguo.org/AnimalTFDB/index.php) [66]. The miRNA family information was from TargetScan (http://www.targetscan.org/vert71/).

Three validation sets of survival analysis were downloaded from Gene Expression Omnibus (GEO, https://www.ncbi.nlm.nih.gov/geo/) [67]. In total, 286 samples in GSE2034 [68], 187 samples in GSE2990 [69] and 198 samples in GSE7390 [70] were analyzed to confirm our results in this study.

### Data filtering and normalization

In order to estimate the quality of data, we counted the number of missing values in DNA methylation data, RNA-seq data and lncRNA data. The sites with missing values in more than 30% samples were removed. Then the average value of the site was calculated to impute the other missing values. In total, 393,983 DNA methylation probes, 3,952 lncRNAs and 16,826 mRNAs were retained.

### DNA methylation of lncRNA

To estimate the methylation level of a given probe, the beta-value was used as the ratio Methy/(Methy + Unmethy), where “Methy” represented the intensity of methylation of the probe and “Unmethy” represented the intensity of unmethylation of the probe [71]. The beta-value “0” stood for unmethylation, and “1” stood for methylation.

We mapped 393,983 450K probes to lncRNA annotation file and extracted the lncRNA promoter information. Since the regulatory mechanism of lncRNA was similar to the PCG in promoter, the region 2kb upstream from the transcription start site (TSS) of lncRNA was regarded as the promoter and the DNA methylation probes in promoter were obtained for further analysis [21, 57]. Then the average value of DNA methylation probes in promoter of one lncRNA was calculated as the DNA methylation level of the lncRNA. The intersection of lncRNAs between the lncRNA methylation profile and the lncRNA expression profile were selected for our further analysis.

### Identification of differentially methylated and differentially expressed genes

Significance analysis of microarrays (SAM) was applied to identify the differential genes between tumor and normal samples for DNA methylation, RNA-seq and lncRNA data, respectively. SAM was developed based on *t-test* and adjusted the *p-value* to assess the statistically significant changes for genes [72]. The differentially methylated lncRNAs were identified with *q*-value < = 0.05 and difference value of DNA methylation level > = 0.1 between tumor and normal samples. The differentially expressed genes were identified with q-value <= 0.05 and fold-change > = 2 or < = 0.5 between tumor and normal samples. The differentially expressed lncRNAs were identified with q-value < = 0.05 [13].

### The prediction of the cis-regulation of lncRNAs

In order to compute the effect of the HMLncs and LMLncs, we used the Genomic Regions Enrichment of Annotations Tool (GREAT: http://bejerano.stanford.edu/great/public/html/index.php) to assess the function of target lncRNAs [73]. The web application used the bed format file of the target lncRNAs to predict the function of HMLncs and LMLncs and tested the false positives of results through the hypergeometric test. The result of annotation consisted of about 20 kinds of ontologies including gene ontology, Human Phenotype, Disease Ontology, MSigDB Cancer Neighborhood, PANTHER Pathway and so on. We extracted the bed format file of lncRNAs from lncRNA annotation file to make the enrichment analysis.

### The construction of the GO term network

The GO term network was picked out from gene2go (18 March 2015 updated), which was downloaded from NCBI [74]. Only considering ‘is_a’ relationships, the final network included 218 terms and 267 edges for HMLncs, 186 terms and 172 edges for LMLncs. The Go term network was visualized through the software Cytoscape [75].

### The prediction of miRNAs targeting lncRNAs

The relationship between miRNAs and diseases was downloaded from the Human MicroRNA Disease Database (HMDD: http://cmbi.bjmu.edu.cn/hmdd), which collected the experimentally supported human miRNA-disease associations.

The hypergeometric test was used to filter the significant functions of the miRNAs with *p*-values less than 0.05:

$$P=1-\sum_{i=0}^{m-1}\frac{C_{M}^{i}C_{N-M}^{n-i}}{C_{N}^{n}}$$ (1)

Where N was the total number of human genome miRNAs in HMDD, M was the size of miRNAs annotated in one disease, n was the size of miRNAs interacted with HMLncs or LMLncs and m was the intersection of n and M.

### The construction of ceRNA network

The interaction pairs of lncRNA-miRNA and mRNA-miRNA were 10,212 and 423,975, respectively. We got 4,563,164 ceRNA pairs between mRNAs and lncRNAs which shared with the same miRNA.

The lncRNA-mRNA ceRNA network was constructed as follows: First, expression correlation between DEGs and DELs was calculated using Pearson correlation coefficient (PCC) between matched mRNA and lncRNA expression profiles:

$$\rho_{ij}=\frac{n\sum_{k=1}^{n}x_{ik}x_{jk}-\sum_{k=1}^{n}x_{ik}\sum_{k=1}^{n}x_{jk}}{\sqrt{n\sum_{k=1}^{n}x_{ik}^{2}-{\left(\sum_{k=1}^{n}x_{ik}\right)}^{2}*\sqrt{n\sum_{k=1}^{n}x_{jk}^{2}-{\left(\sum_{k=1}^{n}x_{jk}\right)}^{2}}}}$$ (2)

Where n was defined as the number of samples in BRCA, x_ik_ was the expression level of gene i in sample k, x_jk_ was the expression level of gene j in sample k.

Because of the competing endogenous interaction between lncRNAs and mRNAs, the lncRNA-mRNA pairs with PCC > 0 and *p-value* < = 0.01 were chosen for further analysis. Second, an lncRNA-mRNA pair which interacted with more than one same miRNA and whose hypergeometric test based on lncRNA-miRNA pair and mRNA-miRNA pair was significant (*p-value* < = 0.01) was considered as a candidate interaction pair (Figure 1). Finally the lncRNA-mRNA pairs satisfying the two conditions were used to construct the lncRNA-mRNA ceRNA network. The ceRNA network was visualized through the software Cytoscape [75].

### Functional enrichment analysis

Function enrichment analysis was performed through the DAVID Bioinformatics Resources (https://david.ncifcrf.gov/) [76]. The DAVID enrichment result only checked KEGG pathways and GO biological process (BP) terms and chose the human genome as the background. The terms with FDR < = 0.05 were considered as the statistically significant result.

### The identification of clinically related mRNAs correlated with lncRNAs

To measure the clinical effect of mRNA modules in ceRNA networks, we used the prognostic index (PI), also named as risk score, to classify the risk groups. The module was consisted of hub mRNAs (degree > = 5) in ceRNA networks. PI was known as the liner Cox model, PI=β_1_x_1_+β_2_x_2_+…+β_p_x_p_, where p was the number of prognostic mRNAs in the module of ceRNA networks, β_p_ was regarded as the risk coefficient which was calculated in the multivariate Cox regression analysis and x_p_ was regarded as the expression level of mRNA_p_. The R package “survival” was used to calculate the risk score of each sample and generated the risk groups. Then the patients were divided into high-risk group and low-risk group by the median value of risk score. Kaplan-Meier method and log-rank test was used to assess the survival difference between two patient groups [13].

## SUPPLEMENTARY MATERIALS TABLES









## Abbreviations

(lncRNAs): long non-coding RNAs
(PCGs): protein coding genes
(CH3): covalent addition of a methyl group
(ORs): odds ratios
(DCIS): ductal carcinoma *in situ*
(IDC): invasive ductal carcinoma
(ncRNAs): non-coding RNAs
(miRNAs): microRNAs
(UTRs): untranslated regions
(Doc): Docetaxel
(BRCA): breast invasive carcinoma
(TCGA): The Cancer Genome Atlas
(HMLncs): high methylated lncRNAs
(LMLncs): low methylated lncRNAs
(GO): gene ontology
(ceRNA): competing endogenous RNA
(GEO): Gene Expression Omnibus
(SAM): Significance analysis of microarrays
(DMLs): differentially methylated lncRNAs
(DELs): differentially methylated lncRNAs
(DEGs): differentially expressed genes
(GREAT): Genomic Regions Enrichment of Annotations Tool
(HMDD): Human MicroRNA Disease Database
(PCC): Pearson correlation coefficient
(K-S): Kolmogorov-Smirnov
(FOX): forkhead box
(IL): Interleukin
(*MAGI2*): membrane associated guanylate kinase
(RE): regulatory element
