# Supplementary material for: The landscape of DNA methylation-mediated regulation of long non-coding RNAs in breast cancer
Source: Oncotarget. 2017 May 8;8(31):51134–50. doi: 10.18632/oncotarget.17705 (PMC5584237; doi:10.18632/oncotarget.17705)
Supplement: Supplementary file 1 [file oncotarget-08-51134-s001.pdf]

The landscape of DNA methylation-mediated regulation of long non-coding RNAs in breast cancer

SUPPLEMENTARY MATERIALS

Supplementary Table 1: The result of cis-regulatory function analysis of lncRNAs. See Supplementary_Table_1

Supplementary Table 2: The result of function enrichment analysis and hallmarks in four ceRNA networks. See Supplementary_Table_2

Supplementary Table 3: The result of miRNA enrichment analysis in four ceRNA networks. The yellow color stands for the breast cancer. See Supplementary_Table_3
